# Narrow safety range of intraoperative rectal irradiation exposure volume for avoiding bleeding after seed implant brachytherapy

**DOI:** 10.1186/1748-717X-7-15

**Published:** 2012-01-31

**Authors:** Ryuji Nakamura, Koyo Kikuchi, Susumu Tanji, Tomonori Yabuuchi, Ikuko Uwano, Satoshi Yamaguchi, Hisanori Ariga, Tomoaki Fujioka

**Affiliations:** 1 Iwate Medical University PET-Liniac Advanced Medical Center, Uchimaru 19-1, Morioka, 020-8505, Japan; 2 Department of Urology, Iwate Medical University School of Medicine, Uchimaru 19-1, Morioka, 020-8505, Japan; 3 Department of Radiology, Iwate Medical University School of Medicine, Uchimaru 19-1, Morioka, 020-8505, Japan; 4 Iwate Medical University Advanced Medical Research, Uchimaru 19-1, Morioka, 020-8505, Japan

**Keywords:** prostate cancer, brachytherapy, dose-volume histogram

## Abstract

**Background & Purpose:**

Rectal toxicity is less common after ^125^I seed implant brachytherapy for prostate cancer, and intraoperative rectal dose-volume constraints (the constraint) is still undetermined in pioneering studies. As our constraint failed to prevent grade 2 or 3 rectal bleeding (bled-pts) in 5.1% of patients, we retrospectively explored another constraint for the prevention of rectal bleeding.

**Materials and methods:**

The study population consisted of 197 patients treated with the brachytherapy as monotherapy using real-time intraoperative transrectal ultrasound (US)-guided treatment at a prescribed dose of 145 Gy. Post-implant dosimetry was performed on Day 1 and Day 30 after implantation using computed tomography (CT) imaging. Rectal bleeding toxicity was classified by CTC-AE ver. 3.0 during a mean 29-month (range, 12-48 months) period after implantation. The differences in rV100s were compared among intraoperative, Day 1 and Day 30 dosimetry, and between that of patients with grade 2 or 3 rectal bleeding (the bled-pts) and of the others (the spared-pts). All patients were divided into groups based on provisional rV100s that were increased stepwise in 0.1-cc increments from 0 to 1.0 cc. The difference in the ratios of the bled-pts to the spared-pts was tested by chi-square tests, and their odds ratios were calculated (bled-OR). All statistical analyses were performed by *t*-tests.

**Results:**

The mean values of rV100us, rV100CT_1, and rV100CT_30 were 0.31 ± 0.43, 0.22 ± 0.36, and 0.59 ± 0.68 cc, respectively. These values temporarily decreased (p = 0.020) on Day 1 and increased (p = 0.000) on Day 30. There was no significant difference in rV100s between the bled-pts and spared-pts at any time of dosimetry. The maximum bled-OR was identified among patients with an rV100us value above 0.1 cc (p = 0.025; OR = 7.8; 95% CI, 1.4-145.8); an rV100CT_1 value above 0.3 cc (p = 0.014; OR = 16.2; 95% CI, 3.9-110.7), and an rV100CT_30 value above 0.5 cc (p = 0.019; OR = 6.3; 95% CI, 1.5-42.3).

**Conclusion:**

By retrospective analysis exploring rV100 as intraoperative rectal dose-volume thresholds in ^125^I seed implant brachytherapy for prostate cancer, it is proved that rV100 should be less than 0.1 cc for preventing rectal bleeding.

## Introduction

When compared to external beam radiotherapy rectal toxicity is less common after ^125^I seed implant brachytherapy for prostate cancer [1], the proximity of the posterior margin of the prostate to the rectal anterior wall compromises the latter's sparing and induces post-implant proctitis, rectal ulcerations, or fistulas, even after prostate seed implant brachytherapy [2,3]. The close correlation between these adverse effects and rectal irradiation exposure volume has been demonstrated by dose-volume histogram (DVH) analysis using computed tomography (CT) images obtained several weeks after brachytherapy. Snyder et al. found a significantly higher frequency of grade-2 proctitis in patients with rectal irradiation exposure volume at a prescribed dose (rV100) greater than 1.3 cc [4]. Waterman et al. also reported that the probability of late rectal morbidity depends both on the dose and on the rectal surface area exposed to 100 Gy radiations [5]. These dose-volume thresholds of the rectal wall could not be adopted as intraoperative constraints for brachytherapy because of discrepancies in dose distribution between intraoperative ultrasound (US)-based and postoperative CT-based dosimetry [6-8]. To date, there appears to be no definitive agreement regarding rV100 constraints during interactive dose assessments. Some investigators have adopted presumptive rV100 constraints, such as 1.0 cc [9], while others have neglected to apply any safe range with rV100 [6].

We initiated seed implantation brachytherapy with a presumptive rV100 constraint of less than 1.0 cc. However, it has since become clear that most patients who develop rectal bleeding have been exposed to an rV100 of less than 1.0 cc. This retrospective study was aimed at exploring the rV100 value thresholds that had been established prospectively, could have prevented the development of rectal bleeding, an infrequent but intractable morbidity resulting from seed implant monotherapy.

## Materials and methods

The study population comprised 197 patients with organ-confined prostate cancer who were treated with ^125^I seed implantation brachytherapy from December 2004 to November 2008 at our hospital, according to a protocol approved by the institutional review board. Every patient provided written consent to participate in the study. Patients who had previously undergone adjuvant external beam radiotherapy were excluded from the study. Patients with high-risk prostate cancer were included in the study when antecedent hormone therapy had successfully controlled the disease for over a year. Patient characteristics are summarized in Table 1. Co-morbidities of the patients included diabetes mellitus (n = 26), hemorrhoids (n = 23), and cardiac disorder necessitated anti-coagulants administration (n = 30). Approximately half of the patients had received hormonal therapy prior to radiotherapy for a median 7.5 months (range, 1-48 months), either as the definitive treatment or as a preparative therapy to achieve volume reduction in unsuitably large prostates.

**Table 1:** Patient characteristics

All patients		
Mean age (y)	66.9	(51-81)
pre-HTx	yes	96
	no	101
T-stage	1c	140
	2a	24
	2b	16
	2c	14
	3	3
Gleason Score	≦6	146
	7	50
	≧8	3
PSA (ng/ml)	< 10	175
	10-20	18
	> 20	4

preHTx, history of hormone therapy before brachytherapy

PSA, prostate-specific antigen

Implantation was performed under spinal anesthesia with the patients reclining in the extended dorsolithotomy position, and a biplanar US probe was positioned in the rectum. Transverse images sliced at a 5 mm distance from the base to the apex of the prostate were captured into the planning system (Interplant version 3.2 CMS Japan; Tokyo, Japan, or Variseed version 7.2; Varian Medical Systems, Palo Alto, CA, USA) for planimetric analysis. The prostate, urethra, and the anterior part of the rectum appeared contoured on each image. Seeds (SourceTech, Bard, USA; 0.338-0.347 mCi) were implanted by transrectal US-guided insertion based on a previously described procedure [10]. The prostate planimetry volume was used to determine the radioactive strength and the number of seeds required. Needles were placed peripherally on the largest prostate slice on the transverse image and loaded with seeds using a Mick applicator (Mick Radionuclear Instruments, New York, USA), under the guidance of sagittal US imaging. Seventy five percent of the seeds were placed at regular intervals along the entire length of the needle within the prostate; the remaining seeds were inserted into the inner needles added to cover the underdosed area, typically located at the base and apex of the prostate gland. Before addition of the inner needles, the US images were re-captured to catch up with prostate swelling and urethral stretching by peripheral needle insertion. All doses were defined using the TG43 formalism [11]. Using simultaneously calculated dose-volume histogram (DVH) parameters, each loading was performed under the following restrictive indices: (i) the dose covering 90% of the prostate volume (pD90) was > 145 Gy; (ii) the prostate volume covered by 100% of the prescribed dose (pV100) was > 95%; (iii) the prostate volume covered by 150% of the prescribed dose (pV150) was < 60%; and (iv) the rectal volume irradiated by 100% of the prescribed dose (rV100) was < 1.0 cc; (v) the urethral volume irradiated by 150% of the prescribed dose was 0 cc. US dosimetric figures were recorded as final results at the end operation. On the day following implantation as well as 30 days post-implantation, the DVH parameters were re-calculated using pelvic CT images obtained with a 3-mm pitch. Rectal preparation was not performed prior to the examination. The same physician delineated both the prostate and the rectum on a series of CT slices. The rectum, which lies posterior to the prostate, was delineated as a solid structure defined by the outer wall, without differentiating the inner wall or contents. Follow-up evaluations after treatment were performed at 3-month intervals for a 5-year period. Routine pre-treatment assessments of rectal mucosal lesions by colonoscopy were not performed. At a mean of 29 months (range, 12-48 months) after the operation (the final follow-up date was June 30, 2010), 28 patients developed rectal bleeding, anywhere from 3 to 24 months after implantation. Of these, 17 of patients had sporadic bleeding, whereas the remaining patients had continual bleeding over several months. Endoscopic assessments were performed for 16 patients and revealed no mucosal changes (n = 11), telangiectasia (n = 2), or radiation proctitis (n = 3). Active mucosal bleeding was treated by photodynamic coagulation hemostasis. Ten rectal bleeding episodes were defined as ≥ grade 2 according to the CTC-AE version 3.0 [12] classifications based on symptoms, medication, or coagulation therapy (the bled-pts). Seven patients showed prostate-specific antigen (PSA) failure defined according to American Society for Therapeutic Radiology and Oncology (ASTRO) guideline [13] during the follow-up period.

### Study Design

#### 1) Comparison of backgrounds between the bled-pts and the others (the spared-pts)

Comparisons between them, using the t-test, were made with regard to the rate of pretreatment hormone therapy, the incidence of co-morbidities, and the mean age at the time of the procedure. The chi-square test was used for comparing the incidence of co-morbidities.

#### 2) Alterations in rV100s at postoperative days 1 and 30

The alterations were evaluated by paired t-tests between intraoperative rV100 (rV100us) and rV100 on Day 1 (rV100CT_1), or rV100CT_1 and rV100 on Day 30 (rV100CT_30). Pearson's test was used to determine any correlations, and linear regression analysis was performed to fit the correlation.

#### 3) Comparison of prostate volume, number of seeds inserted, total radioactivity, and rV100s between bled-pts and spared-pts

At each dosimetric time point, the rV100 rank was compared between them by using a Mann-Whitney *U *test.

#### 4) Likelihood of developing rectal bleeding in association with rV100

For each set of rV100us, rV100CT_1, and rV100CT_30, all patients were divided into 2 groups: those with an rV100 (i) less than or (ii) greater than a provisional value, and the volume was increased from 0 to 1.0 cc stepwise in 0.1-cc increments. The ratio of the number of bled-pts compared with spared-pts was calculated between the 2 groups, and the likelihood of developing rectal bleeding in the group with the larger rV100 was expressed in terms of an odds ratio (the bled-OR). The statistical significance was determined by a chi-square test.

Statistical software SPSS version 11.01.j (SPSS Japan, Tokyo, Japan) was used for data analysis. A difference was regarded as significant if the p value was less than 0.05.

## Results

1): Between the bled-pts and the spared-pts, there were no significant differences in any of expected factors such as age, incidence of co-morbidities, or history of hormone therapy.

2): The mean values for rV100us, rV100CT_1, and rV100CT_30 were 0.31 ± 0.43 cc, 0.22 ± 0.36 cc, and 0.59 ± 0.68 cc, respectively. There was a significant difference between these values. Although positive significant correlations were found between rV100us and rV100CT_1 and between rV100CT_1 and rV100CT_30, the former correlation was as small as 0.142, while the latter was 0.515.

**Figure 1:**
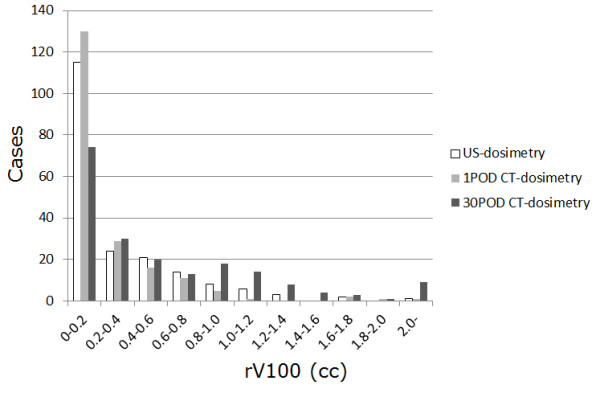
**Histogram of the rV100s for rV100us, rV100CT_1, and rV100CT_30**.

3) The DVH parameters obtained by US-based and CT-based dosimetry on postoperative days (POD) 1 and 30 for all 197 patients are summarized in Table 2. The mean prostate dosage showed reciprocal alterations corresponding to differences in the mean prostate volume; for example, a 23% decrease from US-based to CT-based dosimetry at POD 1 corresponded to a 25% increase in the prostate volume, and an 18% increase from POD 1 to POD 30 on CT-based dosimetry corresponded to a 19% decrease in the prostate volume. Comparison of rV100 histograms in each planning period showed a similar distribution that was partial to the lowest range (0-0.2 cc) (Figure 1). Between the bled-pts and the spared-pts there was no significant difference in the mean values for rV100us (0.43 ± 0.31 vs 0.30 ± 0.43), rV100CT_1 (0.62 ± 0.63 vs 0.20 ± 0.33), rV100CT_30 (0.73 ± 0.42 vs 0.58 ± 0.69) or other dosimetric parameters such as prostate volume (23.8 ± 7.8 cc vs 27.9 ± 7.1 cc), number of seeds (68.3 ± 15.6 vs 73.9 ± 11.9), and total activity (23.2 ± 5.2 mCi vs 25.2 ± 4.0 mCi).

**Table 2:** DVH parameters for each dosimetry period

	**Mean ± s.d**.		
	**intraoperative**	**1st POD**	**30th POD**

pVol (cc)	27.9 ± 7.0	34.4 ± 9.0	27.7 ± 7.2
pD90 (Gy)	179 ± 14.1	137 ± 19.3	160 ± 18.7
pV100 (%)	97.4 ± 2.5	85.8 ± 9.9	92.7 ± 6.0
pV150 (%)	67.6 ± 10.2	44.7 ± 12.4	62.5 ± 13.5
RV100 (cc)	0.31 ± 0.43	0.23 ± 0.36	0.6 ± 0.68
RV150 (cc)	0.03 ± 0.07	0.02 ± 0.06	0.07 ± 0.16

pVol, prostate volume; pD90, dose covering 90% of prostate volume; pV100 and pV150, volume covered by 100% and 150% of the prescription dose, respectively; rV100 and rV150, rectal volume covered by 100% and 150% of prescription dose, respectively

**Figure 2:**
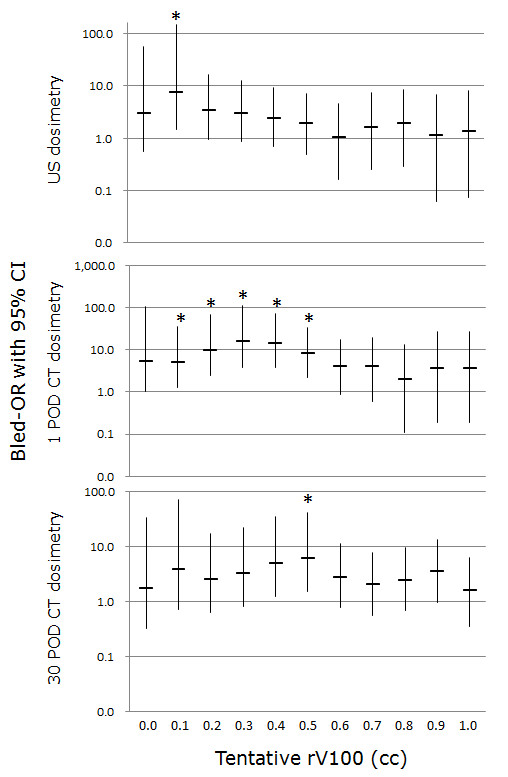
**Bled-ORs with 95% CI led by each provisional rV100 for rV100us, rV100CT_1, and rV100CT_30**. Asterisk indicates significant difference proven by chi-square tests.

4) The bled-ORs for each provisional rV100 value for rV100us, rV100CT_1, and rV100CT_30 are shown in Figure 2. There were considerable differences in the magnitude and corresponding rV100 values of maximally bled-ORs. Maximally bled-ORs with significant chi-square p-values were observed in patients with an rV100us value above 0.1 cc (p = 0.025; OR = 7.8; 95% CI, 1.4-145.8), an rV100CT_1 value above 0.3 cc (p = 0.014; OR = 16.2; 95% CI, 3.9-110.7), and an rV100CT_30 value above 0.5 cc (p = 0.019; OR = 6.3; 95% CI, 1.5-42.3). For rV100CT_1, multiple consecutive high bled-ORs were found, whereas only a single high bled-OR was identified among the rV100s for rV100us and rV100CT_30.

The actual rectal bleeding rate for patients with rV100us less than 0.1 cc is 1.1% while the rate of rV100us greater than 0.1 cc is 8.3%. Differences in DAY 30 prostate dose parameters, such as pD90 (154 ± 19.2 Gy vs 164 ± 17.1 Gy), pV100 (91.1 ± 7.2% vs 94.1 ± 4.5%), and pV150 (59.0 ± 14.9% vs 65.4 ± 11.7%) were significant between the two groups. The rates of PSA failure in both groups were 5/88 and 2/109, respectively, which did not achieve statistical significance.

## Discussion

During seed implant planning, it is frequently difficult to cover the entire prostate with the prescribed dose without exposing the rectum to radiation. As interactive implant planning facilitates fine adjustments with prompt assessment of rectal dose-volume parameters at every seed deposition; therefore, it is worthwhile to establish a reliable intraoperative rV100 threshold to avoid rectal morbidity. This retrospective study showed that, if prostate coverage is prioritized over the sparing of rectal mass, clinicians should be aware of the very narrow safety range regarding the intraoperative rV100, in order to avoid grade 2 rectal bleeding under a prescription dose of 145 Gy.

In this study, the safety range differs by the timing of dosimetry. One of the cause should be the post-implant edema that comes to maximum on Day 1 and decreased thereafter exponentially with the half-life (time for the edema to decrease by 1/2) varied from 4 to 25 days (mean 9.3 days) [14]. As the prostate edema resolved, the distance between the most posterior implanted seeds and the anterior surface of the rectum decreased, and the rectal dose increased exponentially at approximately the same rate the prostate volume decreased [15]. The shift of rV100s partially explains the rectal dose threshold elevation from DAY 1 to DAY 30, but does not that from intraoperative to DAY 1 during which the edema progressed.

It has been reported that rV100us is underestimated in comparison with rV100CT_30. Ishiyama et al. reported that the mean rV100 in 160 patients who underwent ^125^I seed implant brachytherapy on intraoperative US-based dosimetry and CT-based dosimetry on Day 30 was 0.69 cc and 1.02 cc, respectively [7]. Taussky et al. reported that the mean rV100 in 20 patients who underwent ^125^I seed implant brachytherapy increased from 0.07 cc on Day 0 to 0.67 cc on Day 30 when CT-based planning was used [8]. In this study, the rV100CT_30 threshold for rectal bleeding was as low as 0.5 cc. As a result of the underestimation of rV100us and in accordance with these studies, the threshold rV100us was approximated to be zero.

There are some limitations in this study. The bled-OR of the patients with rV100US greater than 0.1 cc displayed a wide CI range. This loose correlation may come from the difficulty to predict rectal bleeding morbidity by DVH analysis of low-dose rate brachytherapy alone, especially if radiation is administered over a period of a couple of months. During this time, the rectal shape will change because of daily defecation and because of the rate of exposure of the anterior wall to full-dose radiation. Merrick et al. compared the rectal dose in CT images obtained 3 months after ^125^I seed implantation between patients with a distended rectum and patients whose rectum was evacuated using an enema; they found that the mean dose for the rectal wall was increased by a factor of 1.5 in the distended state [16]. In addition, Mueller et al. undertook a detailed study of the location of perirectal seeds under the hypothesis that perirectal seeds could be an independent risk factor for rectal morbidity. Using univariate analysis, they found that the number of perirectal seeds is another predisposing factor for higher rectal doses and late rectal bleeding [17]. Further analysis should be performed about these non-dosimetry-related risk factors.

## Conclusion

There is a discrepancy in the rectal dose-volume threshold for post-implant rectal bleeding between the intraoperative and postoperative dosimetry. During ^125^I seed implantation, the rV100 safety range should be set to nearly zero if rectal morbidity is to be avoided.

## Competing interests

The authors declare that they have no competing interests.

## Authors' contributions

RN conceived of the study. KK drafted the manuscripts. ST carried out operation in seed implantation. TY carried out radiotherapy planning in seed implantation. IU performed the statistical analysis. SY made figures. HA and TF participated in its design and coordination. All authors read and approved this manuscript.
